# Mass screening for celiac disease from the perspective of newly diagnosed adolescents and their parents: A mixed-method study

**DOI:** 10.1186/1471-2458-11-822

**Published:** 2011-10-21

**Authors:** Anna Rosén, Maria Emmelin, Annelie Carlsson, Solveig Hammarroth, Eva Karlsson, Anneli Ivarsson

**Affiliations:** 1 Department of Public Health and Clinical Medicine, Epidemiology and Global Health, Umeå University, Umeå, Sweden; 2 Department of Medical Biosciences, Medical and Clinical Genetics, Umeå University, Umeå, Sweden; 3 Department of Clinical Sciences, Social Medicine and Global Health, Lund University, Malmö, Sweden; 4 Department of Clinical Sciences, Pediatrics, Lund University, Lund, Sweden; 5 Norrtälje Hospital, Pediatrics, Norrtälje, Sweden; 6 Växjö Hospital, Pediatrics, Växjö, Sweden

## Abstract

**Background:**

Mass screening for celiac disease (CD) as a public health intervention is controversial. Prior to implementation, acceptability to the targeted population should be addressed. We aimed at exploring adolescents' and parents' experiences of having the adolescents' CD detected through mass screening, and their attitudes towards possible future mass screening.

**Methods:**

All adolescents (n = 145) with screening-detected CD found in a Swedish school-based screening study, and their parents, were invited to this study about one year after diagnosis. In all, 14 focus group discussions were conducted with 31 adolescents and 43 parents. Written narrative was completed by 91 adolescents (63%) and 105 parents (72%), and questionnaires returned by 114 parents (79%). Data were analyzed using qualitative content analysis. In addition, narratives and questionnaire data allowed for quantified measures.

**Results:**

Adolescents and parents described how they agreed to participate *"for the good of others," *without considering consequences for themselves. However, since the screening also introduced a potential risk of having the disease, the invitation was regarded as *"an offer hard to resist." *For the majority, receiving the diagnosis was described as *"a bolt of lightning," *but for some it provided an explanation for previous health problems, and *"suddenly everything made sense." *Looking back at the screening, the predominant attitude was *"feeling grateful for being made aware," *but some adolescents and parents also expressed *"ambivalent feelings about personal benefits." *Among parents, 92% supported future CD screening. The most common opinion among both adolescents and parents was that future CD mass screening should be *"a right for everyone" *and should be offered as early as possible. However, some argued that it should be *"only for sufferers" *with symptoms, whereas others were *"questioning the benefits" *of CD mass screening.

**Conclusions:**

Although the incentives to participate in the CD screening were partly non-personal, and diagnosis was met with surprise, adolescents and parents felt grateful that they were made aware. They welcomed future CD screening, but suggested that it should be conducted earlier in life. Thus, CD mass screening seemed acceptable to most of those who were diagnosed and their parents.

## Background

Mass screening programs seek to identify individuals at risk of or already affected by a disease, and to offer further tests or treatment to reduce the risk of the disease or its complications [1]. Benefits of screening programs should outweigh their possible harm, and the World Health Organization (WHO) has established criteria that should be fulfilled before implementation [2]. These criteria state that the disease should constitute an important health problem with a well understood natural history. In addition, the screening tests, as well as the diagnostic procedure and treatment following a positive result, should be acceptable to the population addressed [3-5]. Population-based screening that targets apparently healthy people raises specific ethical concerns. Individuals invited to a screening should be provided with enough information to make an informed decision as to whether or not to take part [5]. These prerequisites are of even greater importance when the target population comprises minors not entitled to decide by themselves whether or not to participate.

Mass screening might be an option for reducing the negative public health impact of untreated celiac disease (CD) [6]. CD, also called gluten intolerance, is a chronic multi- systemic disorder in which ingestion of gluten triggers an autoimmune reaction resulting in villous atrophy of the small intestine [7]. The disease is a global health problem with an increasing prevalence in many settings. Screening studies in different populations have revealed a CD prevalence varying from 3/1000 to 56/1000, always with most cases being previously undiagnosed [8-12]. Clinically detected CD still may present with gastrointestinal problems, but is increasingly also being diagnosed in patients with subtle feelings of ill health or other extra-gastrointestinal symptoms [13]. Among screening-detected CD patients both symptomatic and asymptomatic cases are found [14,15]. CD is associated with an increased risk for developing short- and long-term negative health consequences such as nutritional deficiencies, anemia, delayed puberty, depression, and low bone mineral density [7]. Serological CD markers with high sensitivity and specificity are available, but a definitive diagnosis requires demonstration of small intestinal histopathologic abnormalities consistent with CD [16]. A strict gluten-free diet, i.e. exclusion of all foods containing wheat, rye or barley, is nearly always an effective treatment that restores the intestinal mucosa and resolves symptoms [7].

Mass screening for CD is controversial, although most of the screening criteria established by the WHO are fulfilled [2,6,17-21]. Health economic evaluations are needed, and the optimal age(s) for testing for CD is yet to be defined. Follow-up studies of screening-detected CD cases are scarce. We recently reported that 54% of adolescents with screening-detected CD perceived improved health one year after diagnosis and initiated treatment [14]. Another follow-up study of screening-detected CD children, diagnosed at 2 to 4 years of age, showed that 10 years later 66% of those following a gluten-free diet experienced improved health [22]. However, we have also shown that the impact of a CD diagnosis on quality of life varies, as changes in perceived health must be balanced against the experiences of living with CD in terms of social sacrifices [14]. Further, other studies have also shown that the lifelong dietary restrictions have considerable effect on daily life and strict compliance is difficult to achieve [23-26].

Before implementation of a CD screening program, its acceptability to the population concerned should be addressed. In a study of 12-year-olds involved in a CD screening we showed that although some children experienced anxiety before receiving the test results, they had or were provided with tools allowing them to cope well and gain confidence [27]. However, no previous study has explored the experiences of those who actually receive a CD diagnosis through screening. Understanding their perspective will help in identifying difficulties associated with this experience and in guiding healthcare providers in their management of these patients. In this study our aim was to explore adolescents' and parents' experiences of having the adolescents' CD detected through mass screening, as well as their attitudes towards possible future mass screening.

## Methods

### Study design

The study utilized a mixed-method design including both qualitative and quantitative methods [28]. First, we explored informants' perceptions and attitudes in focus group discussions. Thereafter we constructed follow-up questionnaires that were sent to the informants and also included an invitation to write a narrative. Transcribed texts from focus group discussions and written narratives were jointly analyzed using qualitative content analysis. In addition, narratives and questionnaire data allowed for quantified measures.

### The setting

A cross-sectional school-based CD screening study of 12-years-olds entitled ETICS - Exploring the Iceberg of Celiacs in Sweden - was performed in five study sites across Sweden [8]. All sixth-graders in the participating schools received an invitation letter containing information about CD and the screening procedure, with one section of the letter specifically addressed to the adolescents, and another to their parents. After obtaining written informed consent from the parents, blood samples from the adolescents were collected at school and analyzed for CD serological markers. If the test results showed elevated CD serological markers, the parent/s received a telephone call from a pediatrician who gave information and offered an appointment within a week at the closest pediatric department. At this appointment further information about CD was given to the adolescent and parent/s and a small intestinal biopsy was recommended. Those with biopsy results confirming a CD diagnosis were recommended to adhere to a lifelong, strict gluten-free diet. Support was offered including consultation with a dietician and follow-up visits to a pediatrician.

The ETICS-study has been described previously [8]. In summary, all adolescents (n = 10041) in all sixth grade school classes in the five study sites were invited. Blood samples from 7208 adolescents (72%, 3467 girls, 3741 boys) without previously detected CD were analyzed for CD serological markers (total s-IgA, antihuman tissue transglutaminase (tTG) of isotype IgA, and if borderline values also for endomysial antibodies). A total of 192 adolescents (2.7%) were found to have elevated levels, and out of those, 180 agreed to undergo a small intestinal biopsy, resulting in 145 screening-detected CD cases (2.0%) diagnosed by biopsy.

### Study population

All adolescents with screening-detected CD found in the ETICS study (n = 145) and their parents constituted the recruitment basis for this study. An overview of the number of informants across the five study sites is given in Table 1. In total, 31 adolescents and 43 parents participated in focus group discussions, 91 adolescents and 105 parents submitted a written narrative, and 114 parents filled in questionnaires. The median age of the adolescents contributing with narratives was 14.6 years (13.9-15.4) and the median time since diagnosis was 15.9 months (11.1-23.2). There were no statistically significant differences in the proportion of girls/boys, the level of mucosal damage of participating and non-participating adolescents or the educational level of participating and non-participating parents (Chi-square-test, p > 0.05).

**Table 1:** Number of informants in the different data collections, across study sites.

**Study site**	**CD-cases**	**Focus group discussion**			**Written narrative**			**Questionnaire**	
			
		**Invited^b^**	**Participated**		**Invited^b^**	**Participated**		**Invited^c^**	**Participated**
			
			**Adolescents**	**Parents **		**Adolescents**	**Parents**		**Parents**
	**n**	**n**	**n**	**n**	**n**	**n**	**n**	**n**	**n**
			
Umeå	16	16	8	9	16	7	10	16	10
Norrtälje	18	18	3	8	18	10	13	18	14
Norrköping	17	17	8	12	17	10	13	17	14
Lund	65	65	12	14	65	45	48	65	55
Växjö	29	0	0	0	29	19	21	29	21
			
**Total**	**145^a^**	**116**	**31**	**43**	**145**	**91**	**105**	**145**	**114**
% females	52	54	45	60	52	53	85	52	84
% of invited	--		21	--^d^		63	72		79

^a ^All screening-detected CD cases found in the multicenter CD screening, forming the recruitment basis for this study.

^b ^Adolescents with their parent(s) were invited.

^c ^Only parents invited.

^d ^Not possible to calculate since we do not know the total number of parents living with the adolescents.

#### Focus group discussions

About one year after diagnosis, all families in four of the five study sites were informed in letters sent to their homes about their possibility to participate in focus group discussions. A few of them immediately replied to confirm their participation. The others were recruited by phone, with the aim to ensure suitable group sizes, and that boys and girls as well as mothers and fathers were represented. In total, 14 focus group discussions were held, involving 31 adolescents and 43 parents. The main reason given for non-participation was lack of time, but a few adolescents also expressed reluctance to talk about their disease. However, parents of this latter group did participate.

#### Follow-up questionnaires

Follow-up questionnaires with invitations to write a narrative were sent to the homes of all adolescents (n = 145) and their parents about one year after diagnosis. Completed narratives were returned by 91adolescents (63%) and 105 parents (72%). Questionnaires regarding future screening were returned by 114 parents (79%).

### Data collection

#### Focus group discussions

Adolescents and parents attended different groups but were mixed in terms of gender. The interviewers (AR, ME, EK) did not have a professional relationship with the informants. A flexible topic guide and hypothetical case stories were used to stimulate the discussions and informants were encouraged to discuss issues of greatest importance to them to increase the likelihood that their own accounts would take priority. The topic guide was structured around the informants' reasoning when deciding to take part in the screening, their experiences of receiving the diagnosis, their reflections concerning their own experience of taking part in a screening, and their attitudes towards CD mass screening. All interviews were digitally recorded. The recorded files were transcribed verbatim by an assistant, and later cross-checked by the first author to ensure accuracy. Transcribed texts were entered into the software Open Code [29].

#### Follow-up questionnaires

##### Short reflective narratives

Along with answering follow-up questionnaires, both the adolescents and their parents were asked to write individual narratives. They were encouraged to reflect on their overall experience of the CD screening and specifically to elaborate both on how they felt about receiving the diagnosis, and on their recommendations about possible future CD screening. To facilitate confidentiality between adolescents and parents, they were instructed to return the questionnaires in separate envelopes. The length of the narratives ranged from one to two handwritten pages. All narratives were transcribed verbatim and entered into the software Open Code.

##### Questions on future screening

The parental questionnaire included two questions that were utilized in this study: *i) *whether a CD screening should be implemented, with pre-determined response alternatives (yes, no, don't know), and *ii) *at what age a screening should be conducted, which was an open-ended question.

### Qualitative analysis

**Figure 1:**
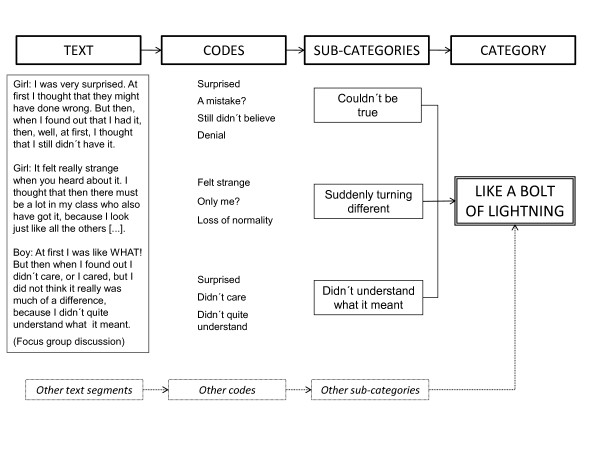
**The process of qualitative content analysis, moving from text to an interpretation of the abstract meaning**.

Transcribed texts from all focus group discussions and written narratives were jointly analyzed systematically with the aim of gaining understanding and interpreting the meaning of the text. For this purpose we used qualitative content analysis as described by Graneheim and Lundman [30]. Initially the texts were read several times to obtain a flexible frame of reference. Thereafter, all texts were labeled with codes to conceptualize and categorize the informants' experiences. Coded data related to the different content areas were gathered and compared regarding similarities and differences. Codes sharing communalities were grouped into sub-categories, which later supported the constructed categories, reflecting more abstract levels of the meaning in the informants' accounts. As we aimed at capturing and presenting the variation in the data, we kept close to the informants' own descriptions of their experiences. An illustration of the qualitative analytical process is presented in Figure 1. The analysis, done by the first author but continuously discussed with the co-authors, was characterized by constant comparison of the sub-categories and categories with the original text to ensure that the interpretations were grounded in the data.

### Quantitative analysis

As all adolescents and parents were invited to write narratives it allowed us to quantify the proportions supporting the categories derived from the qualitative analysis. Each individual narrative was reviewed to judge which of the categories within each content area that were supported, or not reflected upon. In addition we analyzed questionnaire data on parents' attitudes towards future screening. For analysis of quantified measures, SPSS 19.0 (SPSS Inc., Chicago, IL) was used.

### Ethical considerations

When the adolescents were invited to the focus group discussions, they were informed that participation was voluntary. It was also emphasized that they should only share things they felt comfortable about sharing. After the sessions, a pediatrician from the research group was available to answer questions from both adolescents and parents. Written informed consent was obtained from caregivers of all participating adolescents. The Regional Ethical Review Board in Umeå, Sweden, approved the study [Dnr UmU 04-156M].

## Results

**Figure 2:**
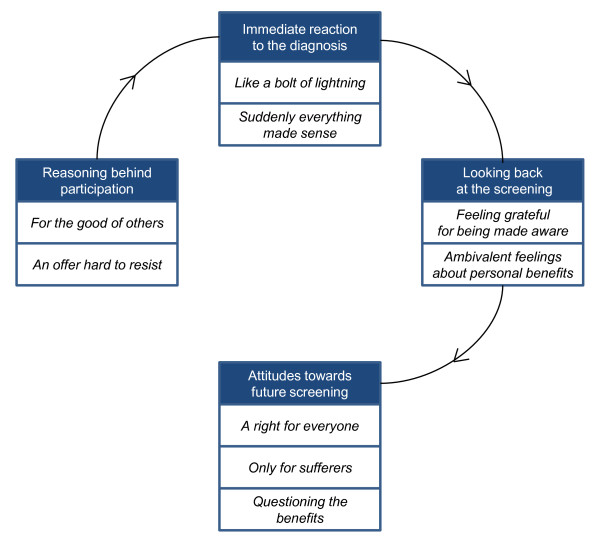
**Content areas and categories derived from qualitative analysis reflecting experiences of, and attitudes towards, CD-screening**.

The qualitative analysis of focus group discussions and written narratives resulted in nine categories that corresponded to four different content areas: reasoning behind participation in the screening, immediate reactions to the diagnosis, looking back at the screening, and attitudes towards future mass screening (Figure 2).

In the following text we describe results of the qualitative analysis in more detail by highlighting the content areas (headings) with the corresponding categories (subheadings). Quotations from focus group discussions and written narratives show how our interpretations are grounded in the data. As a complement, Table 2 gives the proportions of adolescents and parents who in their narratives supported the categories developed for the different content areas. In addition, results from the questionnaire for parents regarding their opinions about future CD screening are presented in Table 3.

**Table 2:** Quantification of informants who in their narratives supported the categories derived from the qualitative analysis.

Content areas with related categories^a^	Adolescents	Parents
	n (%)	n (%)
Immediate reaction to the diagnosis		
Like a bolt of lightning	68 (75%)	73 (70%)
Suddenly everything made sense	5 (5%)	19 (18%)
*Not reflected upon *	*18 (20%)*	*13 (12%)*

Looking back at the screening		
Feeling grateful for being made aware	35 (38%)	76 (72%)
Ambivalent feelings about personal benefits	9 (10%)	8 (8%)
*Not reflected upon*	*47 (52%)*	*21 (20%)*

Attitudes towards future screening		
A right for everyone	62 (68%)	72 (68%)
Only for sufferers	8 (9%)	2 (2%)
Questioning the benefits	9 (10%)	8 (8%)
*Not reflected upon*	*12 (13%)*	*23 (22%)*

Total	91 (100%)	105 (100%)

^a ^This table does not include content area Reasoning behind participation, since that was only asked for, and reflected upon, in the focus group discussions.

**Table 3:** Parental questionnaire data on attitudes towards a future celiac disease screening.

Question	Response-alternative	Responses	
		n	(%)
Do you think a general screening for celiac disease should be implemented in the future?	Yes	105	(92%)
	No	2	(2%)
	Don't know	7	(6%)

If a screening is implemented, at what age do you think it should take place?	0 - 5 years	34	(30%)
	6-10 years	39	(34%)
	> 10 years	12	(11%)
	Don't know	24	(21%)
	Non-response	5	(4%)

**Total**		114	(100%)

### Reasoning behind participation in the screening

#### For the good of others

The focus group discussions revealed that the reason for participating was mainly based on a willingness to contribute to research, without considering potential personal implications. Both adolescents and parents expressed that they considered participation their responsibility to help others and that they generally trusted that they would be treated fairly in research projects.

"When it [the invitation] came it was the most natural thing, you just had to do it for research."

Mother, FGD

"My mother told me I should take the test. She said we had to show them someone who is healthy. That didn't work very well."

Girl, FGD

#### An offer hard to resist

Some adolescents also related that, the blood sampling itself became a way of proving themselves. They described that some classmates who refused the test were looked upon as ridiculous, but it also seemed that refusal to participate was considered a more active way of behaving than following the crowd. Thus, peer pressure probably influenced decision-making among the adolescents. Whereas some adolescents described how they decided for themselves to participate, others revealed that their parents decided for them, with little option for them to refuse.

" I thought I would just do it for the sake of doing it, also almost just to be in the project."

Boy, FGD

Both adolescents and parents described that they did not expect the test to be positive, with the exception of one mother who already had a daughter with CD and suspected that her son also suffered from it. However, once the screening was offered and a potential risk of having the disease was introduced, an element of insecurity was created. This insecurity, which was more prominent among parents than among adolescents, led to wanting reassurance about not suffering from the disease, and made the offer to participate in the screening hard to resist.

"We usually contribute. In part we thought it would be easy to get this reassurance, since we didn't believe we would end up here. But I suppose it's good to have got it [CD] confirmed, and it might also help other people."

Father FGD

### Immediate reaction to the diagnosis

#### Like a bolt of lightning

For the majority, news of the diagnosis was perceived as a bolt of lightning, illustrating that the screening test, previously considered incidental or even forgotten about, abruptly played a big role in their lives. Some revealed that when they received the first test results they doubted that they were correct. Another common feeling that arose was that the diagnosis suddenly transformed the adolescent from being normal to being different. Parents described how they felt that the screening took their healthy child away from them.

"It felt really strange when I heard about it. I thought that then there must be a lot in my class who also have it, because I look just like all the others. So it felt kind of strange. Have they really tested my blood samples?"

Girl, FGD

A pediatrician gave the test results to the parents first, by phone, and the parents then were messengers who gave the results to the adolescents. The adolescents described this as an awkward situation, because neither they nor their parents understood what it really meant. This lack of knowledge fostered anxiety among both parents and adolescents.

" [When receiving the results] I wasn't totally sure either, but I had a little hope that maybe it wasn't so, but what was it then? Something even worse, I thought. THEN I was scared about that and searched the internet and got nightmares that it was something even worse."

Mother, Narrative

Some adolescents described how in retrospect they felt betrayed by the information given before the test, as they thought it had not sufficiently prepared them for the consequences of participating in the screening. They described feelings of regret at having decided to participate, or being disappointed in their parents who decided for them. Words like "being caught" or "getting stuck" were frequently used when they described receiving the diagnosis. They also emphasized that more explicit information about the consequences of the screening should be given before the test.

#### Suddenly everything made sense

Adolescents experiencing health problems before diagnosis, as well as their parents, described how the diagnosis came as a relief. Some of them had sought healthcare without CD being detected, and now with the diagnosis they found out what the underlying problem was.

"We'd been to the pediatric clinic earlier for different diffuse problems, so when we found out about this, it was as if it suddenly dawned on me."

Father, FGD

Overall, boys and girls seemed to have similar reactions towards receiving the diagnosis. They shared numerous descriptions of how they reacted with anger, anxiety, fear and sadness. Both adolescents and parents related that what was hardest to handle was the fact that there was no cure. However, mothers also expressed feelings of guilt for not having suspected that their adolescent suffered from a disease. Interestingly, only mothers, not fathers, expressed having a guilty conscience.

"Our daughter had begun to feel worse before the study. She was pale, short of breath, tired and depressed. We understood that something was wrong, but when we received the celiac diagnosis we were still surprised, because she hadn't had big problems with her stomach. As a mother and a nurse, I had a really guilty conscience because I hadn't understood earlier."

Mother, Narrative

### Looking back at the screening

#### Feeling grateful for being made aware

Knowledge of a previously undetected diagnosis was perceived as important in itself and adolescents and parents expressed gratitude for being made aware. However, the reasons differed depending on the adolescents' perceived health before the screening. If they had been experiencing symptoms, becoming aware of the diagnosis gave them a means of feeling better. However, adolescents without previous symptoms, as well as their parents, expressed that the screening was even more important to them, as otherwise they would not have known about the disease. Having no suspicions and then realizing that their adolescent had the disease led to feelings of insecurity and worry about what could have happened if they had not been in the screening.

"You're happy when it's detected. Since she wasn't sick, it's even better that we found out now. It could have gone on forever."

Mother, narrative

Both adolescents and parents had adopted the doctor's argument and were concerned about future complications. Interestingly, the complications mentioned by the informants differed in the different study sites. In some sites the greatest concern was getting diabetes, whereas in others cancer was mentioned as the complication of most concern.

"I think knowing is only positive. I think it would be worse not knowing, and risk developing all those complications."

Father, FGD

Having internalized the risk for future complications functioned as a strong motivator for adhering to the gluten-free diet. When asked to compare the dietary recommendations with other (more general) health promoting advice, the adolescents described how the dietary recommendations became more personal, and thereby more important to follow.

Girl A: "I think that you're more motivated to eat gluten-free food than not to start smoking, because smoking still is your own choice. I mean that to eat gluten-free is like, it's just best for me. I mean you almost have to do it."

Boy B: "Well, if someone tells you to quit smoking you don't take it so seriously. Of course you shouldn't smoke, you know that, but if you get the recommendation to eat gluten-free food, then it's more personal. At least that's what I think."

*Girl C: "It sort of feels more important"*.

FGD

#### Ambivalent feelings about personal benefits

Some adolescents and parents were ambivalent about the benefits of adhering to the dietary recommendations. This attitude was associated with not experiencing any health improvement and/or being ambivalent about whether health complications really would occur if they did not comply with the diet. This was also related to concern about the social consequences of having to adhere to a strict diet and to beliefs about a higher threshold for the amount of gluten that could be tolerated. However, even when doubt was expressed about the benefits of complying with the diet, the uncertainly of not knowing for sure if there would be future consequences led to ambivalent feelings about the value of the diagnosis and adhering to the treatment.

"I got very annoyed when my doctor called and said that I was gluten intolerant, not because I was gluten intolerant, but because I had no symptoms. When we said that to a dietitian, she said that I should at least try gluten-free food for three months, and I did, but I didn't feel better [...] Personally, I don't think you need to be tested for something you don't suffer from!"

Girl, Narrative

### Attitudes towards future screening

From the parental questionnaire data we found that 92% of the responding parents wanted a CD screening to be implemented, whereas 2% were against it and 6% answered that they did not know (Table 3). The majority of parents (64%) wanted a screening to be conducted during the period from 0-10 years of age. The analysis of the qualitative data shed light on the reasoning behind the attitudes of both the adolescents and the parents.

#### A right for everyone

This category, reflecting the most common standpoint towards future CD mass screening among both adolescents and parents, was strongly related to having internalized the risk of possible negative outcomes of untreated CD. It reflects the opinion that if society knows how to find the disease, it is a human right to be offered the test. The adolescents expressed a concern for people who were still unaware of their disease and considered it unfair that the screening study only comprised sixth graders in certain cities instead of being offered to everyone. Parents, on the other hand, described how they could not understand other parents not letting their adolescents participate in the screening. There were also personal reasons for welcoming a screening for everyone. The adolescents hoped that if more cases were found, the availability of gluten-free products would increase as would efforts by scientists to find a cure.

" I think you should have a screening, because if it turns out that more children have it, then maybe scientists will make some effort so that you don't have to have it."

Boy, narrative

Thus, the main argument for offering the test to everyone was that it was important for people to become aware. This response was sometimes based on the informant's experience of improved health after treatment was initiated, and therefore the wish that others would have the same possibility, whereas other informants believed that a screening was even more important for those without symptoms, since screening would be the only way to identify their disease. Their opinions seemed related to a general trust that a CD screening would only be offered if it involved a greater good for those involved.

"It would be good to test every child, because if they don't feel good, then they will be able to feel better. That's how it was for me."

Girl, Narrative

"I think that people should be part of it [the screening] because you have nothing to lose. It's good to know so that you don't get sick in the future."

Boy; FGD

A predominant opinion was that if a future CD screening is implemented, it should be conducted earlier in life. Adolescents thought that it would have been easier to adjust to the gluten-free diet if the CD had been detected earlier, so that they would not have become accustomed to 'all the good food' containing gluten. However, some advocated that the screening should take place in the teenage period, arguing that a young person needs to be mature enough to manage the transition from being normal to becoming 'a celiac', a transition experienced as a big step in life. Parents, on the other hand, advocated screening as early in life as possible with the hope of more effectively avoiding negative consequences of undetected disease. They were concerned that the untreated disease might influence the child's growth and development more negatively if diagnosed during or after puberty. However, parents also related that they perceived the teenage period as being filled with so many other commitments that it would be easier to adjust to the dietary recommendations if the diagnosis were obtained earlier. Some said that the timing of the current screening (at 12 years of age) was the worst possible age.

#### Only for sufferers

Some adolescents emphasized that although it is important to offer a screening test, only those suffering from symptoms should take part. This attitude was related to not experiencing any short-term benefits of the treatment themselves and a belief that testing everyone would be superfluous and expensive for society.

"I took the test but didn't have any symptoms. Now I have to eat gluten-free. I just think that only those who feel bad should be tested."

Boy, Narrative

#### Questioning the benefits

Some adolescents and parents were unsure about the potential of a future screening. They were not convinced about the scientific basis for recommending a screening or the benefits of adhering to the treatment in the long run. Adolescents could also be indecisive because of their own experience of finding it hard to adhere to the treatment when they had no immediate health benefits, and they did not want others to experience the same difficulties.

"Yes, I think it might be good to test all children for celiac disease. But on the other hand, that depends if it turns out that there are some (like me for example) who would have been able to eat normally and still not get any complications, and who would needlessly eat a gluten-free diet for the rest of their lives that wouldn't be good."

Girl, Narrative

## Discussion

This is, to our knowledge, the first study to explore how adolescents and their parents experience having the adolescent's CD detected through mass screening, as well as their opinions about future screening efforts.

The mixed-method study design allowed for an in-depth understanding of adolescents' and parents' reasoning, as well as quantified measures of the representativeness of some of the findings in the study population. Focus group discussions build on group interaction and can facilitate sharing experiences, especially when attempting to elicit children's views [31]. The emergent design of our study implied that the analysis from the first focus group discussions helped to steer the discussions towards a deeper exploration of details in the following groups. Preliminary analysis of focus group discussion data guided the triangulation of data collection methods by suggesting a need for including narratives, as well as short-answer questions in follow-up questionnaires.

A limitation of our study is that we did not gain approval from the ethical review board to invite those who declined participation in the screening and those whose serological marker results were positive but who declined further clinical investigation, a group likely to be more negative towards mass screening than our informants. Another limitation is that our study does not allow for exploration of differences between experiences of receiving a screening-detected CD diagnosis in a research study compared to a general screening program or being diagnosed due to symptoms. It is likely that the context of being invited to a screening *study *influenced both decision-making and reactions to the diagnosis in a way that routine screening offered within the healthcare system may not. The quantified measures of the proportions of individual narratives supporting different categories should of course be interpreted with caution, since the informants might support a certain view without having expressed it in the narratives. However, these figures do provide information concerning what the informants spontaneously brought up in their accounts.

We found that the reasons adolescents and parents participated in the screening involved a feeling of duty to contribute to research as well as a means of handling the risk they were introduced to. Their idea that participation would lead to greater benefits for everyone most likely reflects societal norms to help others. These findings are in line with results found in a previous qualitative ETICS sub-study involving adolescents who had not yet received their test results [27]. However, in that study the adolescents felt involved, well informed and perceived that they had a general understanding about CD. Contrary to this, we found that some adolescents in retrospect felt not having participated in the decision to take part in the screening. They also felt that they had not been provided with enough information about the possible consequences of their participation and expressed a need for improved information before the test. These results imply that it is probably first when diagnosed that the consequences are fully incorporated and detailed information is sought. Nevertheless, this underlines the importance of true informed consent, which is especially challenging when approaching minors and their guardians. Our findings emphasize the importance of involving children and adolescents in the information and consent process, even if the final decision is made by their guardians. However, the information provided when inviting persons to CD screening programs must be balanced to avoid unnecessary anxiety while waiting for the test results, since the majority of those participating in the screening will not be diagnosed with the disease.

Being surprised, feeling angry and sad, or even questioning if the test results were correct characterized the initial reaction to the diagnosis. Later these feelings developed into a belief in the benefits of becoming aware. The emotional responses of denial, anger, and later acceptance correspond to stages of grief first described by Elisabeth Kübler-Ross [32]. As most adolescents and parents had no suspicion about the disease, they felt unprepared. Lack of sufficient preparation for receiving "bad news" has also been observed in studies of other screening-detected diagnoses [33,34]. Having a moderate amount of concern prior to a stressful event (proactive coping) is suggested to facilitate the psychological adaptation once the event occurs [35]. As a screening-detected diagnosis is often unexpected, and the transition from being healthy to becoming a patient is abrupt, this probably influences the psychological adaptation process negatively. Healthcare providers therefore need to be sensitive to what screening-detected patients actually know about their disease, and how they cope with the diagnosis, in order to provide adequate support and follow-up care. However, in our study we also found that for some informants, mainly parents, the diagnosis actually decreased anxiety and was perceived as a relief, since it provided an explanation for the symptoms that were experienced. These results are supported by other studies showing that among screening-detected CD cases true symptomatic cases are also found [15,36]. Overall, boys and girls seemed to have similar reactions towards receiving the diagnosis. However, we found that mothers, compared to fathers, were more prone to express feelings of guilt for not previously having suspected what their child was suffering from. This gender difference in parental coping has been observed in other studies [37,38].

The screening seemed acceptable to most of the adolescents and their parents. They felt grateful for becoming aware, based on experiences of improved health after initiated treatment or an adaptation of the risks of what otherwise could have happened. In fact, those who were asymptomatic before diagnosis, and their parents, seemed even more prone to advocate screening, as it would be the only way to identify their disease. We also observed that the individual doctor's view of risk strongly influenced the informants since the risks mentioned differed between study sites. The informants' accounts reflect how the screening itself produced concerns, resulting in an increased risk awareness during the screening process. Implementation of screening programs in society as a means of preventing complications could be seen as part of the modern risk society described by Beck [39]. In our study, some informants had ambivalent feelings about the personal benefits. According to Beck this can be seen as a consequence of the modern risk society where increased awareness of risk is followed by an increased capability to question the suggested risks. It is often people themselves who start to reflect on risks, since it become harder to rely on scientists to do this for them.

A common argument against CD screening is that strict compliance with a gluten-free diet is hard to achieve in symptomatic CD patients, and even harder in those presumed to be asymptomatic [18,40]. Indeed, we found that experiencing health improvements after initiating the gluten-free diet functioned as a motivator for adherence. However, even if the adolescent's health had not improved, most adolescents and parents expressed that avoiding future health complications motivated dietary compliance. In addition, adhering to the diet was perceived as more important than following health promotion messages aimed at society as a whole, since CD was perceived as more of a personal threat.

Both adolescents and parents welcomed future CD screening but thought it should be implemented earlier than in the present screening. Previous studies have shown that compliance with a gluten-free diet among adolescents is higher when CD is diagnosed earlier in life due to symptoms [41]. However, a crucial factor in deciding the optimal age (or ages) involves obtaining a balance between identifying the majority of cases, and not having to repeat the screening many times. It is now evident that CD can develop at any age; however, the age distribution for disease initiation (sero-conversion) is not yet known. Thus, multi-country birth cohort studies with repeated blood-sampling for analysis of CD serological markers are needed, and such studies are underway [42].

In our study, some informants questioned the benefits of the CD screening and asked for more scientific evidence about the consequences of exposing the population to a mass screening. However, most adolescents and parents responded that if there are means to find the disease, then everyone should have the right to be offered the test. Screening to identify undiagnosed CD was seen as beneficial for health outcomes, but the diagnosis was also important in itself. The latter finding relates to the ethical considerations introduced by genetic screening tests that are increasingly advocated, where preventive measures are sometimes not available [43]. If information about a diagnosis (or carrier status) is alone viewed as a right and as sufficient for advocating screening, some of the currently accepted principles for screening will be ruled out. Thus, the challenge with mass screening is that the presumed "right to know" must be balanced against the principle of non-maleficence, and the autonomy of individuals with a right to choose *not *to know.

Before implementing a mass screening for CD we need to ensure that the potential benefits outweigh the harm (and costs) both for involved individuals and society. Many of the suggested mass screening criteria are fulfilled for CD, but not yet all of them. Further research is needed to evaluate the extent to which treatment of screening-detected CD reduces long-term negative health consequences. In addition, health economic evaluations are needed specifically for CD mass screening, but also allowing for comparison with other public health interventions. We have taken a first step to increase the understanding of CD screening acceptability from the perspective of newly diagnosed adolescents and their parents in a Swedish context. We believe that some of our results are transferable to screenings in other settings, and for other diseases. Nevertheless, additional studies on the acceptability of CD screening are needed that involve other age groups and cultural settings, as well as those individuals found to have normal and false positive results in a screening.

## Conclusions

This study contributes increased understanding concerning the acceptability of a CD screening among Swedish adolescents with CD detected through screening and their parents. Although the incentive to participate was found to be non personal benefits, and the diagnosis was met with surprise, the most predominant reaction was a feeling of gratitude for being made aware of the diagnosis. Although some adolescents and parents were more reluctant regarding screening, the most predominant view was that screening for everyone would be welcomed, with the argument that if we know how to detect the disease, being offered the test is a human right.

## List of abbreviations

CD: celiac disease; WHO: World Health Organization; ETICS: Exploring the Iceberg of Celiacs in Sweden

## Competing interests

The authors declare that they have no competing interests.

## Authors' contributions

AR contributed to the design of the study, collected data, performed data analyses and wrote the first and final drafts. AI and ME contributed to the design of the study and guided the data collection, analysis and writing of the manuscript. ME and EK assisted in the data collection. All authors read and revised the drafts critically, and read and approved the final version.

## Pre-publication history

The pre-publication history for this paper can be accessed here:

http://www.biomedcentral.com/1471-2458/11/822/prepub

## References

[B1] Holland WW Stewart S Screening in Disease Prevention: What works? 2005 Oxon: Radcliffe Publishing Ltd

[B2] Wilson JMG Jungner G Principle and practice of screening for disease 1968 Geneva: World Health Organization

[B3] Frankenburg WK Selection of Diseases and Tests in Pediatric Screening Pediatrics 1974 54 (5):612 616 4453461

[B4] Marshall KG Prevention. How much harm? How much benefit? 3. Physical, psychological and social harm CMAJ 1996 155 2 169 176 8800074 PMC1487962

[B5] Marshall KG Prevention. How much harm? How much benefit? 4. The ethics of informed consent for preventive screening programs CMAJ 1996 155 4 377 383 8752062 PMC1488063

[B6] Mearin ML Ivarsson A Coeliac disease: is it time for mass screening? Best Pract Res Cl Ga 2005 19 3 441 452 10.1016/j.bpg.2005.02.004 15925848

[B7] Di Sabatino A Corazza GR Coeliac disease Lancet 2009 373 9673 1480 1493 10.1016/S0140-6736(09)60254-3 19394538

[B8] Myleus A Ivarsson A Webb C Danielsson L Hernell O Hogberg L Karlsson E Lagerqvist C Norstrom F Rosen A Celiac disease revealed in 3% of Swedish 12-year-olds born during an epidemic J Pediatr Gastroenterol Nutr 2009 49 2 170 176 10.1097/MPG.0b013e31818c52cc 19516192

[B9] Csizmadia CGDS Mearin ML von Blomberg BME Brand R Verloove-Vanhorick SP An iceberg of childhood coeliac disease in the Netherlands Lancet 1999 353 9155 813 814 10459972 10.1016/S0140-6736(99)00243-3

[B10] Catassi C Ratsch IM Gandolfi L Pratesi R Fabiani E El Asmar R Frijia M Bearzi I Vizzoni L Why is coeliac disease endemic in the people of the Sahara? Lancet 1999 354 9179 647 648 10.1016/S0140-6736(99)02609-4 10466670

[B11] Fasano A Berti I Gerarduzzi T Not T Colletti RB Drago S Elitsur Y Green PHR Guandalini S Hill ID Prevalence of celiac disease in at-risk and not-at-risk groups in the United States - A large multicenter study Arch Intern Med 2003 163 3 286 292 10.1001/archinte.163.3.286 12578508

[B12] Maki M Mustalahti K Kokkonen J Kulmala P Haapalahti M Karttunen T Ilonen J Laurila K Dahlbom I Hansson T Prevalence of celiac disease among children in Finland New Engl J Med 2003 348 25 2517 2524 10.1056/NEJMoa021687 12815137

[B13] Telega G Bennet TR Werlin S Emerging new clinical patterns in the presentation of celiac disease Arch Pediatr Adolesc Med 2008 162 2 164 168 10.1001/archpediatrics.2007.38 18250242

[B14] Rosen A Ivarsson A Nordyke K Karlsson E Carlsson A Danielsson L Hogberg L Emmelin M Balancing health benefits and social sacrifices: A qualitative study of how screening-detected celiac disease impacts adolescents' quality of life BMC Pediatr 2011 11 32 10.1186/1471-2431-11-32 21569235 PMC3120678

[B15] Hoffenberg EJ Emery LM Barriga KJ Bao F Taylor J Eisenbarth GS Haas JE Sokol RJ Taki I Norris JM Clinical features of children with screening-identified evidence of celiac disease Pediatrics 2004 113 5 1254 1259 10.1542/peds.113.5.1254 15121938

[B16] Hill ID Dirks MH Liptak GS Colletti RB Fasano A Guandalini S Hoffenberg EJ Horvath K Murray JA Pivor M Guideline for the diagnosis and treatment of celiac disease in children: recommendations of the North American Society for Pediatric Gastroenterology, Hepatology and Nutrition J Pediatr Gastroenterol Nutr 2005 40 1 1 19 10.1097/00005176-200501000-00001 15625418

[B17] Logan RF Screening for coeliac disease--has the time come for mass screening? Acta Paediatr Suppl 1996 412 15 19 8783749 10.1111/j.1651-2227.1996.tb14241.x

[B18] Kumar PJ European and North American populations should be screened for coeliac disease Gut 2003 52 2 170 171 10.1136/gut.52.2.170 12524394 PMC1774950

[B19] Hoffenberg EJ Should all children be screened for celiac disease? Gastroenterology 2005 128 4 Suppl 1 S98 103 15825134 10.1053/j.gastro.2005.02.023

[B20] Evans KE McAllister R Sanders DS Should we screen for coeliac disease? No BMJ 2009 339 b3674 10.1136/bmj.b3674 19762414

[B21] Fasano A Should we screen for coeliac disease? Yes BMJ 2009 339 b3592 10.1136/bmj.b3592 19762413

[B22] van Koppen EJ Schweizer JJ Csizmadia CG Krom Y Hylkema HB van Geel AM Koopman HM Verloove-Vanhorick SP Mearin ML Long-term health and quality-of-life consequences of mass screening for childhood celiac disease: a 10-year follow-up study Pediatrics 2009 123 4 e582 588 10.1542/peds.2008-2221 19336349

[B23] Jadresin O Misak Z Sanja K Sonicki Z Zizic V Compliance with gluten-free diet in children with coeliac disease J Pediatr Gastroenterol Nutr 2008 47 3 344 348 10.1097/MPG.0b013e31816f856b 18728532

[B24] Hopman EG Koopman HM Wit JM Mearin ML Dietary compliance and health-related quality of life in patients with coeliac disease Eur J Gastroenterol Hepatol 2009 21 9 1056 1061 10.1097/MEG.0b013e3283267941 19209068

[B25] van Doorn RK Winkler LM Zwinderman KH Mearin ML Koopman HM CDDUX: a disease-specific health-related quality-of-life questionnaire for children with celiac disease J Pediatr Gastroenterol Nutr 2008 47 2 147 152 10.1097/MPG.0b013e31815ef87d 18664865

[B26] Roma E Roubani A Kolia E Panayiotou J Zellos A Syriopoulou VP Dietary compliance and life style of children with coeliac disease J Hum Nutr Diet 2010 23 2 176 182 10.1111/j.1365-277X.2009.01036.x 20163513

[B27] Nordyke K Myleus A Ivarsson A Carlsson A Danielsson L Hogberg L Karlsson E Emmelin M How do children experience participating in a coeliac disease screening? A qualitative study based on children's written narratives Scand J Public Health 2010 38 4 351 358 10.1177/1403494810368454 20413585

[B28] Creswell JW Fetters MD Ivankova NV Designing a mixed methods study in primary care Ann Fam Med 2004 2 1 7 12 10.1370/afm.104 15053277 PMC1466635

[B29] Umdac Open Code (version 3.4) 2007 Umeå: Umdac & Epidemiology and Global Health, Department of Public Health and Clinical medicine, Umeå University

[B30] Graneheim UH Lundman B Qualitative content analysis in nursing research: concepts, procedures and measures to achieve trustworthiness Nurs Educ Today 2004 24 2 105 112 10.1016/j.nedt.2003.10.001 14769454

[B31] Heary CM Hennessy E The use of focus group interviews in pediatric health care research J Pediatr Psychol 2002 27 1 47 57 10.1093/jpepsy/27.1.47 11726679

[B32] Kübler-Ross E On death and dying 1969 New York: MacMillan Publishing

[B33] Bratt EL Ostman-Smith I Sparud-Lundin C Axelsson BA Parents' experiences of having an asymptomatic child diagnosed with hypertrophic cardiomyopathy through family screening Cardiol Young 2010 1 7 10.1017/S1047951110001290 20883596

[B34] Peel E Parry O Douglas M Lawton J Diagnosis of type 2 diabetes: a qualitative analysis of patients' emotional reactions and views about information provision Patient Educ Couns 2004 53 3 269 275 10.1016/j.pec.2003.07.010 15186863

[B35] Aspinwall LG Taylor SE A stitch in time: Self-regulation and proactive coping Psychol Bull 1997 121 3 417 436 9136643 10.1037/0033-2909.121.3.417

[B36] Johnston SD Watson RG McMillan SA Sloan J Love AH Coeliac disease detected by screening is not silent--simply unrecognized QJM 1998 91 12 853 860 10.1093/qjmed/91.12.853 10024951

[B37] Gray DE Gender and coping: the parents of children with high functioning autism Soc Sci Med 2003 56 3 631 642 10.1016/S0277-9536(02)00059-X 12570979

[B38] Schwab R Gender differences in parental grief Death Stud 1996 20 2 103 113 10.1080/07481189608252744 10160537

[B39] Ritzer G Sociological theory 2000 5 Singapore: McGraw-Hill Companies

[B40] Whitaker JK West J Holmes GK Logan RF Patient perceptions of the burden of coeliac disease and its treatment in the UK Aliment Pharmacol Ther 2009 29 10 1131 1136 10.1111/j.1365-2036.2009.03983.x 19245681

[B41] Hogberg L Grodzinsky E Stenhammar L Better dietary compliance in patients with coeliac disease diagnosed in early childhood Scand J Gastroenterol 2003 38 7 751 754 10.1080/00365520310003318 12889562

[B42] Hogen Esch CE Rosen A Auricchio R Romanos J Chmielewska A Putter H Ivarsson A Szajewska H Koning F Wijmenga C The PreventCD Study design: towards new strategies for the prevention of coeliac disease Eur J Gastroenterol Hepatol 2010 22 12 1424 1430 21389794 10.1097/MEG.0b013e32833fe9ae

[B43] Stone DH Stewart S Screening and the new genetics; a public health perspective on the ethical debate J Public Health Med 1996 18 1 3 5 8785072 10.1093/oxfordjournals.pubmed.a024458

